# Biodegradable Quercetin-Incorporated Poly(Lactic Acid)/Chitosan Functional Films: A Study of the Properties and Application in Enhancing Fish Preservation

**DOI:** 10.3390/foods14162771

**Published:** 2025-08-09

**Authors:** Xiaolu Li, Si Wu, Tao Feng, Shijing Wu, Weiwen Xu, Qingmiao Wang, Yu Wang, Ning Hu, Xiaowen Shi

**Affiliations:** 1 Hubei Key Laboratory for Efficient Utilization and Agglomeration of Metallurgic Mineral Resources, College of Resources and Environment Engineering, Wuhan University of Science and Technology, Wuhan 430081, China; 2 School of Resource and Environmental Science, Wuhan University, Wuhan 430079, China

**Keywords:** poly(lactic acid), chitosan, quercetin, active packaging, fish freshness

## Abstract

Traditional plastic packaging materials have brought serious environmental pollution and a number of health risks; so the development of biodegradable polymers as an alternative has received increasing attention. Here, active packaging materials with antioxidant, antimicrobial, and biodegradable properties were prepared using poly(lactic acid) (PLA) and chitosan loaded with quercetin. The experimental results demonstrate that the PLA/chitosan/quercetin film achieved an impressive ABTS radical scavenging rate of up to 98.2%, and the inhibition rates against Gram-negative (*E. coli*) and Gram-positive (*S. aureus*) bacteria were 87.60% and 80.45%, respectively. Furthermore, the composite film exhibited excellent oxygen barrier properties and biodegradability. Shelf life tests demonstrate that the PLA/chitosan/quercetin film retarded fish spoilage by 2 days compared to commercial polyethylene film. Additionally, the color changes in the film showed significant correlation with fish freshness, serving as an effective freshness indicator. Therefore, the PLA/chitosan composite film containing quercetin has a good application prospect in fish preservation and intelligent monitoring of fish freshness.

## 1. Introduction

As a necessity in daily life, packaging materials perform vital functions in food preservation. Commonly used packaging materials such as polyethylene (PE) and polypropylene (PP) are mostly made of fossil-derived polymers and additives [1]. Some toxic substances may migrate from these polymers and additives into food over time. These plastics are difficult to degrade in the environment, and potential carcinogens such as bisphenol A can diffuse from the polymer into water and the environment, exerting deleterious effects on humans and the environment [2]. Meanwhile, with the growing focus on food quality and safety, active packaging materials have gained widespread attention. Currently, three predominant types of active packaging are employed for fresh food preservation: antimicrobial, antioxidant, and moisture control, aiming to enhance the quality of fresh food and prolong storage stability [3]. Therefore, developing eco-friendly active packaging is important for the environment and food safety.

Poly(lactic acid) (PLA) is primarily fabricated from bio-based raw materials such as corn and sugarcane through fermentation and polymerization processes and has good thermal stability and biodegradability [4]. Meanwhile, PLA exhibits excellent optical transparency and moderate physical toughness, and it is relatively low-cost. As a result, it can serve as a replacement for some conventional petroleum-based plastics [5,6]. Furthermore, research on the functional modification of PLA is continually progressing. For instance, chitosan-modified PLA can enhance its bioactivity, offering new possibilities for the commercial application and functional diversity of PLA.

Chitosan is a biodegradable polymer with unique biocompatibility; it is antibacterial, has low toxicity, and so on [7]. As a result, chitosan polymers have found extensive applications in food packaging, agriculture, biomedicine, etc. [8]. However, pure chitosan films are brittle, and their antioxidant and antimicrobial properties are limited during application [9,10,11]. Polymer blending technology can enhance the structural and technological attributes of materials, particularly in enhancing tensile strength and improving thermal stability, etc. This technology has gradually become an important way of enabling material innovation [12]. To optimize the mechanical properties and antimicrobial and antioxidant performance of chitosan films, many studies have mixed plant extracts and auxiliary polymers with them to form active composite films [13,14], which not only confer versatility to food packaging materials, but also contribute to the extension of shelf life.

Phenolic compounds are naturally synthesized through plant secondary metabolism, which can inhibit reactive oxygen species and nitrogen [15]. Quercetin is a natural polyphenol abundantly found throughout various fruits and vegetables. It possesses diverse bioactive properties, such as antioxidative, antiviral, and antimicrobial capabilities, and has been extensively used in medicine and food packaging [16,17].

In this work, a bioactive PLA/chitosan/quercetin composite film was prepared, and the optimal ratio was determined by the study of mechanical and antioxidant properties. Furthermore, the PLA/chitosan/quercetin film was applied to preserve crucian using PE film and the PLA/chitosan film as controls. Thiobarbituric acid reactive substance (TBARS) accumulation, total volatile base nitrogen (TVB-N) production, total viable count (TVC) proliferation, and pH fluctuations were sequentially analyzed over a period of 9 days, and the changes in the PLA/chitosan/quercetin film were recorded. In conclusion, the PLA/chitosan/quercetin film is beneficial to enhance the shelf life of fish for refrigerated preservation and demonstrates potential for intelligent food packaging applications in response to the freshness of fish.

## 2. Materials and Methods

### 2.1. Materials

Chitosan (Mw = 4.06 × 10^5^ g/mol, 85% deacetylation value) was acquired from Tokyo Chemical Industry (TCI, Shanghai, China). Quercetin was bought from Sigma-Aldrich (St. Louis, MO, USA). Poly(lactic acid) (PLA, Mw = 7.77 × 10^4^ g/mol) was acquired from Nature Works 2003D (Blair, NE, USA). Boric acid, ABTS (2,2′-Azinobis(3-ethylbenzothiazoline-6-sulfonic acid ammonium salt)), EDTA (ethylenediaminetetraacetic acid), trichloroacetic acid (TCA), thiobarbituric acid (TBA), and Span 80 were purchased from Aladdin (Shanghai, China). Formic acid (88%), chloroform (99.8%), N, N-Dimethylformamide (DMF, 99.5%), potassium persulfate, ascorbic acid, magnesium oxide, methyl red, bromothymol blue, anhydrous ethanol, sodium chloride, beef extract, and agar were obtained from Sinopharm chemical reagent Co., Ltd. (Shanghai, China). Tryptone was obtained from Oxoid, Ltd. (Hampshire, UK). Aseptic homogenization bags were purchased from Hunan Bickman Holdings Co., Ltd. (Changsha, China). Crucian was bought at the farmers’ market. All reagents were of analytical grade and were used without further purification.

### 2.2. Preparation of the PLA/Chitosan/Quercetin Film

Chitosan was dissolved in formic acid solution (5 g/L) and stirred with a magnetic stirrer at room temperature (25 °C). PLA was dissolved in a chloroform solution with stirring (20 g/L). This solution was next added dropwise to an equal volume of DMF solution to prepare a 10 g/L PLA solution [18]. After mixing evenly, 2% of Span 80 was introduced to the solution. The PLA solution was stirred at 60 °C and 1000 rpm, and the chitosan solution was incorporated into the PLA solution at 0.145 mL/min to prepare a PLA/chitosan mixed solution (PLA: chitosan ratios of 6:3, 6:4, and 6:5), which were then cast into Petri dishes and oven-dried at 60 °C for 24 h. Then, anhydrous ethanol was poured over the film to facilitate the complete removal of the film, resulting in a PLA/chitosan film. The PLA/chitosan film was soaked in a quercetin solution (200, 500, and 800 mg/L in anhydrous ethanol) for 24 h, maintaining a film to solution ratio of 1.5% (*w*/*v*). Then, it was dried at 25 °C to obtain the PLA/chitosan/quercetin film. The thickness of the film was calculated as 0.03 mm.

### 2.3. Characterization

#### 2.3.1. Mechanical Strength

The universal tensile testing machine (CMT6350, Shenzhen SANS Testing Machine Co. Ltd., Shenzhen, China) was utilized to test the mechanical properties [19]. Each film specimen (1 × 5 cm) was tested under the following conditions: 5 mm/min crosshead speed with 28 mm initial gauge length.

#### 2.3.2. Morphological and Structural Characterization

Film surfaces were examined by scanning electron microscope (SEM, TESCAN MIRA LMS, Brno, Czech) at different magnifications. The structure of the samples was analyzed by a Fourier transform infrared (FTIR) spectrometer (INVENIO R, Ettlingen, Germany) in transmittance mode with 16 scans at 4 cm^−1^ resolution over the scanning range of 4000–400 cm^−1^.

#### 2.3.3. Water Contact Angle (WCA)

The WCA values of the PLA/chitosan and PLA/chitosan/quercetin film were quantitatively characterized using a contact angle measuring instrument (German OCA25, Filderstadt, Germany). The static contact angle was using a drop of deionized water (10 μL) from a micro-syringe, and the WCA values were recorded at 10 s [20].

#### 2.3.4. Water Vapor Permeability (WVP)

The WVP test was adopted from Gulzar et al. [21] with minor modifications. An amount of 10 g of color-changing silica gel was placed in a beaker, and the beaker was sealed with the PLA/chitosan film and the PLA/chitosan/quercetin film. Samples were stored in a humidity-controlled chamber (75% RH, maintained by saturated NaCl solution) at 25 °C. The beakers were weighed every hour for 10 h continuously. Three parallel samples were taken for each group. The WVP and water vapor transmission rate (WVTR) values of the PLA/chitosan and PLA/chitosan/quercetin films were derived from Equations (1) and (2) [22].

(1) $$\mathrm{WVP}=\;\frac{∆W\times a}{∆t\times A\times ∆P}$$

(2) $$\mathrm{WVTR}=\;\frac{∆W}{∆t\times A}$$

where ΔW, A, a, Δt, and ΔP represent the mass water vapor transmitted (g), the effective membrane area (m), the average membrane thickness (m^2^), the testing time (s), and the water vapor pressure difference across the film (1583.7 Pa), respectively.

#### 2.3.5. Oxygen Transmission Rate (OTR)

The OTR measurement was based on the GB/T 1038.1-2022 [23] standard and conducted utilizing the Labthink VAC-V2 differential pressure gas permeability tester. The testing was conducted under controlled environmental conditions (23 ± 2 °C, 49 ± 1% RH).

#### 2.3.6. Antibacterial Activity

Antibacterial properties of the PLA/chitosan/quercetin film against *E. coli* and *S. aureus* were quantitatively assessed employing the dilution spread plate method [24]. Experimental protocols are available in the Supplementary Data. The inhibition rate was calculated as follows:

(3) $$\mathrm{Inhibition}\;\mathrm{rate}\;(\%)\;=\;\frac{A}{A_{0}}\times 100\%$$

where A represents the colony count of the experimental group containing the film, and A_0_ indicates the colony count of the blank control without the film.

#### 2.3.7. Biodegradability

To evaluate the biodegradability, the PLA/chitosan and PLA/chitosan/quercetin film modified with 500 mg/L quercetin (PLA: chitosan ratio of 6:3) were prepared in triplicate. Using PE films as controls, all three types of films (55 mm diameter) were buried 10 cm deep in natural soil. Photographs of the films were taken, and their areas were calculated using image software (Adobe Photoshop 2024, Version 25.0.0.37). The degradation rate was calculated using Equation (4) [25]. The soil pH and moisture content were measured with reference to HJ 962-2018 [26] and HJ 613-2011 [27], respectively.

(4) $$\mathrm{Degradation}\;\mathrm{rate}\;(\%)\;=\;\frac{A_{0}-A_{t}}{A_{0}}\times 100\%$$

Here, the initial film area (A_0_) and degraded film area (Aₜ) represent the measurements before and during degradation.

### 2.4. Antioxidant Activity

To measure the antioxidant capacity of the film, the ABTS solution (7 mM) was blended with potassium persulfate and placed in the dark for 14 h to generate ABTS^+^ [28]. After being diluted 100 times, the absorbance of the solution was recorded as A_0_ (blank). The sample film was placed in 3 mL ABTS diluted solution and reacted at 25 °C for 1 h. For detailed steps, see the Supplementary Data. The formula for the ABTS radical scavenging rate is shown in Equation (5):

(5) ABTS Scavenging (%) = (A_0_ − A_1_)/A_0_ × 100%

where A_0_ is the absorbance of the diluted solution and A_1_ is that of the diluted solution containing the film.

### 2.5. Shelf Life Tests of Crucian During Refrigerated Storage

#### 2.5.1. Pre-Treatment of Fresh Crucian Samples

The fresh crucian samples were processed immediately after procurement. First, 20 g fish was weighed and put into a polypropylene (PP) tube (Figure S1). Then, each tube was sealed with the PE, PLA/chitosan, and PLA/chitosan/quercetin films, respectively, and subjected to refrigeration (4 °C). The parameters of the fish were measured daily for 9 days, and the process for each parameter was performed in triplicate.

#### 2.5.2. pH

From each film-sealed tube, 2 g fish was taken and soaked in 20 mL ultrapure water. The solution was stirred at high speed for 2 min using a mixer, followed by 30 min equilibration before pH measurement.

#### 2.5.3. TBARS

Malondialdehyde (MDA) quantification was performed using the spectrophotometry method [29]. At first, a 7.5% trichloroacetic acid (TCA) solution (which contains 0.1% EDTA) and a 0.02 mol/L thiobarbituric acid (TBA) solution were prepared. Next, 5 g fish was taken from each tube, minced, and placed into a beaker containing 25 mL TCA and allowed to stand for 3 min. Aliquots (10 mL) of supernatant were reacted with equal volumes of TBA solution at 90 °C for 40 min. After cooling, the mixtures were centrifuged (1600 rmp, 5 min). Then, chloroform (5 mL) was added to the extract. Absorbance at 532 nm and 600 nm was measured by UV-Vis spectrophotometer. The calculation formula for TBARS was as follows:

(6) TBARS (mg MDA/kg) = (A_532_ − A_600_/155) × 72.06

where A_532_ and A_600_ represent the absorbance values measured at 532 nm and 630 nm, respectively. The value 155 indicates the optical density of the MDA solution (1 mmol/L) recorded at 532 nm. The value 72.06 represents the molecular weight of MDA.

#### 2.5.4. TVB-N

According to the national standard GB/T 5009.228-2016 [30], the TVB-N value of the crucian sample was determined using an automatic Kjeldahl nitrogen analyzer (Hanon Instruments K1160, Jinan, China). Detailed procedures are described in the Supplementary Data.

#### 2.5.5. TVC

The evaluation of fish shelf life commonly relies on microbial testing methodologies [29]. Initially, a solid medium was prepared using beef extract, peptone, agar, sodium chloride, and ultrapure water. Physiological saline was prepared using 0.9% sodium chloride. Next, 5 g fish was put into an aseptic homogenization bag containing 45 mL of sterile physiological saline. The homogenization bag was tapped for 3 min. The obtained samples were diluted with physiological saline by 10 times, followed by an appropriate gradient dilution. Finally, 100 μL of the diluted solution was spread on the plate and incubated at 37 °C for 48 h to observe colony counts. All materials required sterilization processing.

### 2.6. Color Analysis of the PLA/Chitosan/Quercetin Film

The color changes in the PLA/chitosan/quercetin film were photographed and recorded. The film was then analyzed in detail using Photoshop to quantify its L*, a*, and b* values, where the L* indicated lightness, ranging from 0 (black) to 100 (white). The a* indicated green (−) or red (+), and b* indicated blue (−) or yellow (+). Total color difference ∆E [31] and the yellowness index YI [32] were derived from Equations (7) and (8), respectively:

(7) $$\Delta E=\sqrt{{{(L^{*}-L}_{0})}^{2}+{{(a^{*}-a}_{0})}^{2}+{{(b^{*}-b}_{0})}^{2}}$$

(8) $$\mathrm{YI}\;=\;\frac{142.86b^{*}}{L^{*}}$$

where L_0_, a_0_, and b_0_ are the original color values of the film. L*, a*, and b* are the color values of the film at different days.

### 2.7. Data Analysis

Statistical analysis was performed using one-way ANOVA followed by Duncan’s multiple range test (SPSS 27.0), with significance defined as *p* < 0.05.

## 3. Results and Discussion

### 3.1. Characterization of the PLA/Chitosan/Quercetin Film

#### 3.1.1. Mechanical Properties

**Figure 1:**
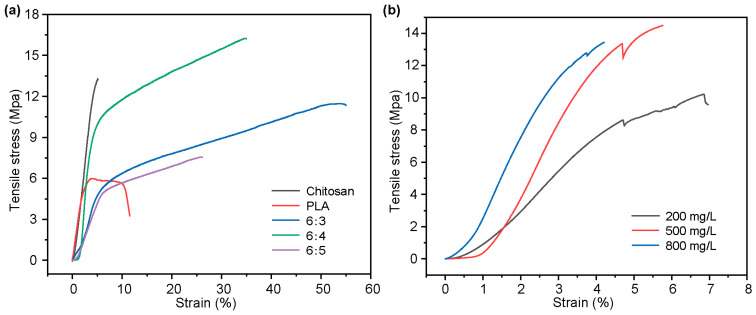
(**a**) Stress–strain curves of the chitosan, PLA, and PLA/chitosan films (PLA: chitosan ratios of 6:3, 6:4, and 6:5). (**b**) Stress–strain curves of the PLA/chitosan/quercetin films modified with 200, 500, and 800 mg/L quercetin (PLA: chitosan ratio of 6:3).

The blending of PLA and chitosan is beneficial to enhance its mechanical performance, and the optimal ratio of PLA and chitosan blending was investigated by stress–strain curves. Figure 1a indicated that the elongation at break of pure chitosan film reached 5% when tensile stress (TS) was 13.2 MPa. In contrast, pure PLA exhibited good plasticity under the same test conditions, with an elongation at break of 11.5%. At a 6:3 PLA/chitosan ratio, the composite film achieved optimal mechanical properties with 55% elongation at break and 11.3 MPa tensile strength, which could be attributed to the interaction between PLA and chitosan through hydrogen bonding and van der Waals forces, creating a stable interface in the composite film [33]. Therefore, the ratio of PLA to chitosan was chosen to be 6:3 in the subsequent experiments.

The mechanical properties of the PLA/chitosan/quercetin films modified with 200, 500, and 800 mg/L quercetin were next investigated. Figure 1b demonstrate that compared with the PLA/chitosan film, the addition of quercetin significantly enhanced the tensile strength of the PLA/chitosan/quercetin film, reaching a maximum value of 14.49 MPa at a 500 mg/L concentration with no further improvement observed at higher concentrations. However, the elongation at break of the composite film showed a decreasing trend with increasing quercetin loading concentration, which is in agreement with prior studies [34]. This was possibly ascribed to the interactions between the hydroxyl groups in quercetin and the PLA and chitosan, which enhanced the mechanical strength of the material, but also limited the extension of the polymer molecular chains [35].

#### 3.1.2. Antioxidative Properties

**Figure 2:**
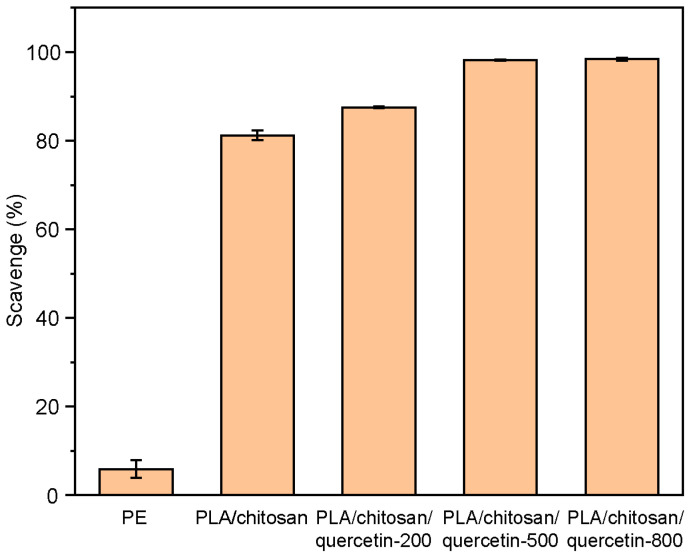
Antioxidant properties of the PE, PLA/chitosan, and PLA/chitosan/quercetin films modified with 200, 500, and 800 mg/L quercetin (PLA: chitosan ratio of 6:3).

The development of active packaging materials with efficient antioxidant functions is of significant application value to prolong the shelf life of products. Quercetin is a bioflavonoid with antioxidant properties [2], which contains phenolic hydroxyl groups that scavenge free radicals [36,37]. Using PE film as a comparison, the ability of PLA/chitosan films and PLA/chitosan/quercetin films to scavenge ABTS free radicals was studied in Figure 2. PE film only had a weak scavenging ability (5.8%), whereas the PLA/chitosan film demonstrated a significant scavenging rate of 81.2%. Notably, the scavenging rate increased gradually with increasing quercetin concentration and peaked at 98.2% with a quercetin concentration of 500 mg/L, after which it stabilized. Thus, the quercetin concentration was determined to be 500 mg/L.

#### 3.1.3. FTIR Analysis

**Figure 3:**
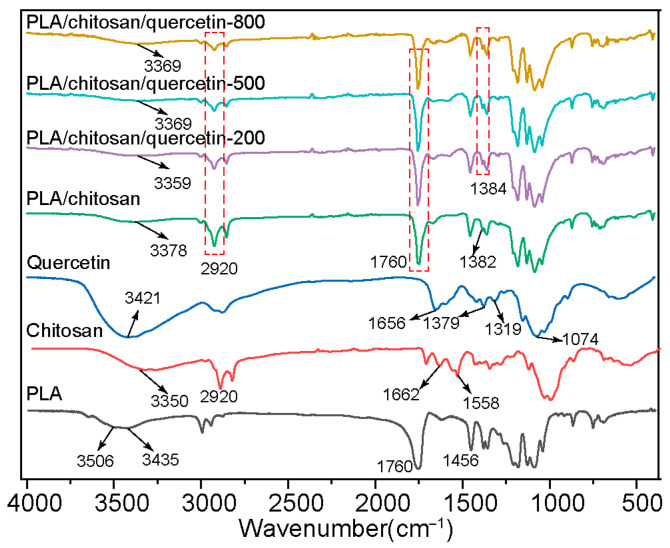
FTIR spectra of pure PLA, chitosan, quercetin, PLA/chitosan film, and PLA/chitosan/quercetin films modified with 200, 500, and 800 mg/L quercetin (PLA: chitosan ratio of 6:3).

The structure and composition of PLA, chitosan, quercetin, and the PLA/chitosan/quercetin film were investigated using FTIR, as shown in Figure 3. For PLA, the broad peaks around 3506 and 3435 cm^−1^ represented the stretching vibrations of O-H and N-H, and the characteristic peak at 1760 cm^−1^ represented the stretching vibration of C=O [38,39]. The asymmetric stretching of C-H appeared around 1456 cm^−1^ [40]. For chitosan, the broad peak at 3350 cm^−1^ was ascribed to the overlap of O-H and N-H stretching vibrations, and 2920 cm^−1^ was for C-H stretching vibration [41]. The peaks of its amide groups appeared near 1662 cm^−1^ (amide I band, C=O stretching vibration) and 1558 cm^−1^ (amide II band, N-H bending vibration) [19,42]. For quercetin, the broad band around 3421 cm^−1^ was caused by hydroxyl groups, and the peak at 1379 cm^−1^ was due to the O–H bending vibration of phenolic groups [34].The peaks at 1319 and 1074 cm^−1^ represented C-O-C anti-symmetrical and symmetrical stretching [36].

In the PLA/chitosan composite film, the O-H vibration was observed at 3378 cm^−1^, and the amide I band of chitosan disappeared, while the amide II band weakened. At the same time, the C=O stretching vibration of PLA (1760 cm^−1^) and the C-H stretching vibration of chitosan (2920 cm^−1^) were also reflected in the PLA/chitosan film, indicating non-covalent interactions between PLA and chitosan molecules [2]. After loading different concentrations of quercetin into the PLA/chitosan film, the O-H stretching vibration peak in the composite film further shifted to lower wavenumbers, possibly due to the formation of new hydrogen bonds between quercetin and PLA/chitosan [34]. Additionally, the characteristic peak that appeared at 1384 cm^−1^ was assigned to the O-H bending vibration of quercetin, displaying a minor shift compared to the characteristic peak of pure quercetin at 1379 cm^−1^. This observation further verified the successful incorporation of quercetin.

#### 3.1.4. Morphology and Surface Properties

**Figure 4:**
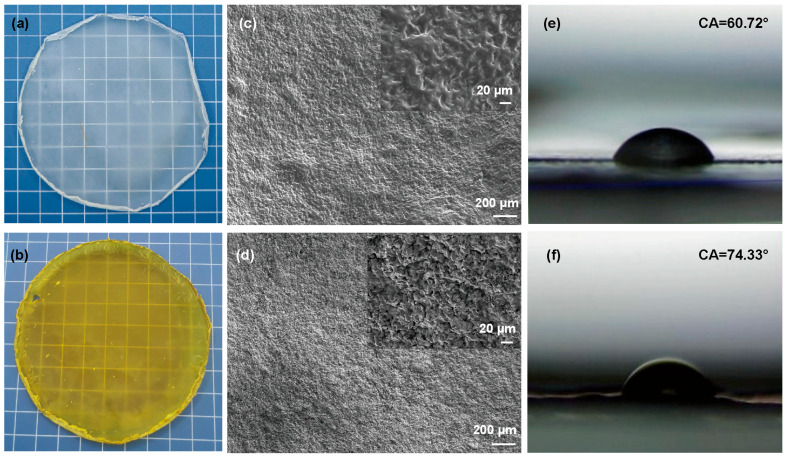
Transparency of the (**a**) PLA/chitosan and (**b**) PLA/chitosan/quercetin film. SEM images of the (**c**) PLA/chitosan and (**d**) PLA/chitosan/quercetin film. WCA of the (**e**) PLA/chitosan and (**f**) PLA/chitosan/quercetin film. (PLA: chitosan ratio of 6:3; quercetin concentration was 500 mg/L).

Figure 4a shows that the PLA/chitosan film had high transparency, while the incorporation of quercetin resulted in a yellow coloration of the composite film (Figure 4b). The presence of quercetin also had a remarkable effect on the film’s microstructure due to the interaction with the polymer. The SEM photographs (Figure 4c) show that the PLA/chitosan film surface was relatively even and dense, while the surface of the PLA/chitosan/quercetin film was relatively rough with some crystal gaps, but the overall distribution was uniform, as shown in Figure 4d. These results indicate that quercetin had good compatibility with polymers, but it was also accompanied by a small amount of aggregation and microscopic separation [43,44]. According to the Wenzel model, the increase in surface roughness usually results in the increase in WCA. Figure 4e shows that the WCA of the PLA/chitosan film was 60.72°, possibly due to the presence of numerous hydrophilic groups in chitosan, such as hydroxyl (-OH) and amino (-NH^2^) groups [45]. Figure 4f demonstrates that the WCA of the PLA/chitosan/quercetin film reached 74.33°, which might be caused by the hydrophobicity of quercetin and the microstructural changes in the film [46].

#### 3.1.5. Barrier Properties

**Figure 5:**
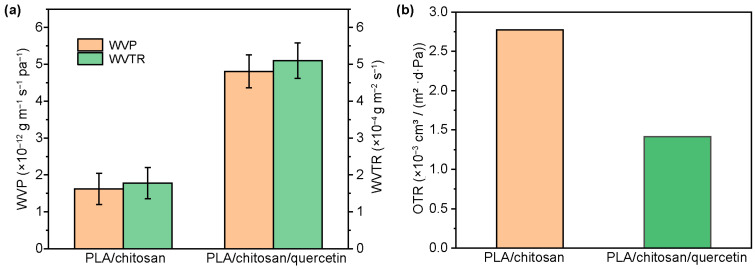
(**a**) WVP and WVTR of the PLA/chitosan and PLA/chitosan/quercetin films. (**b**) OTR of the PLA/chitosan and PLA/chitosan/quercetin films. (PLA: chitosan ratio of 6:3; quercetin concentration was 500 mg/L).

WVTR and OTR are important performance indicators for food packaging films. Good water vapor permeability can help maintain the moisture within food, while the appropriate oxygen transmission rate helps extend the shelf life of the food. Previous research showed that although the introduction of quercetin increased the WCA of the film (Figure 4f), it also led to the formation of discontinuous phases between polymers (Figure 4d), which increased the film’s surface porosity and weakened water vapor barrier performance [47]. This observation aligned with the findings of this study. Figure 5a demonstrates that compared to the PLA/chitosan film, the WVTR of the PLA/chitosan/quercetin film increased from 1.78 ± 0.42 to 5.10 ± 0.48 (10^−4^ g m^−2^ S^−1^), accompanied by an increase in water vapor permeability. Although quercetin increased the film’s surface porosity, the quercetin molecules optimized the internal structure via hydrogen bonding interactions with the PLA/chitosan matrix [48]. This interaction reduced the oxygen transmission path, lowering the oxygen permeability from 2.772 × 10^−3^ cm^3^/(m^2^·d·Pa) for the PLA/chitosan film to 1.416 × 10^−3^ cm^3^/(m^2^·d·Pa) for the PLA/chitosan/quercetin film (Figure 5b).

### 3.2. Antibacterial Activity Analysis

**Figure 6:**
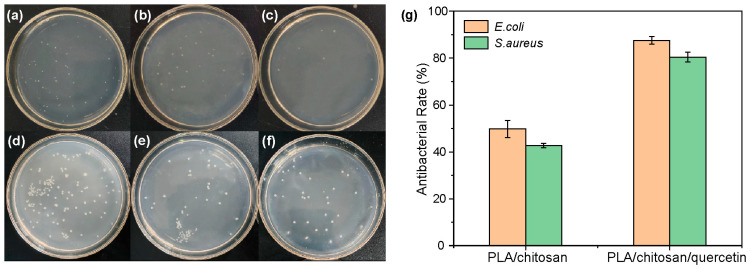
Antibacterial activity of composite film against *E. coli*: (**a**) blank control, (**b**) the PLA/chitosan film, and (**c**) the PLA/chitosan/quercetin film. Antibacterial activity of composite film against *S. aureus*: (**d**) blank control, (**e**) the PLA/chitosan film, and (**f**) the PLA/chitosan/quercetin film. (**g**) Antibacterial rate of the PLA/chitosan and PLA/chitosan/quercetin films against *E. coli* and *S. aureus.* (PLA: chitosan ratio of 6:3; quercetin concentration was 500 mg/L).

The antibacterial effect on bacteria is a key indicator to assess the performance of food packaging films. This study compared the inhibition effects of the PLA/chitosan and PLA/chitosan/quercetin films on *E. coli* and *S. aureus* (Figure 6 and Figure S2). Compared with the blank controls in Figure 6a,d, the numbers of *E. coli* (Figure 6b) and *S. aureus* (Figure 6e) were reduced after treatment with the PLA/chitosan film, and the inhibition rates reached 49.82% and 42.73%, respectively (Figure 6g). After treatment with the PLA/chitosan/quercetin film, the numbers of *E. coli* and *S. aureus* in Figure 6c,f were further reduced, and the inhibition rate increased to 87.60% and 80.45%, accordingly (Figure 6g). The increased inhibition rate for these two types of bacteria likely results from quercetin’s release, which damages cell membrane structures or interferes with key metabolic pathways within microbial cells [49].

### 3.3. Application of the PLA/Chitosan/Quercetin Film

The polyunsaturated fatty acids present in crucian are susceptible to the action of free radicals and reactive oxygen species, leading to lipid oxidation and hence oxidative stress. Furthermore, microbial activities cause nitrogenous substances such as proteins and amino acids in fish to decompose into small molecules such as ammonia and trimethylamine, resulting in spoilage and nutrient loss. To examine the freshness retention capability of the PLA/chitosan/quercetin film in preserving crucian, the TBARS, TVB-N, pH, and TVC values of the fish wrapped with these films were measured over a 9-day period. PE film and the PLA/chitosan film were utilized as controls.

**Figure 7:**
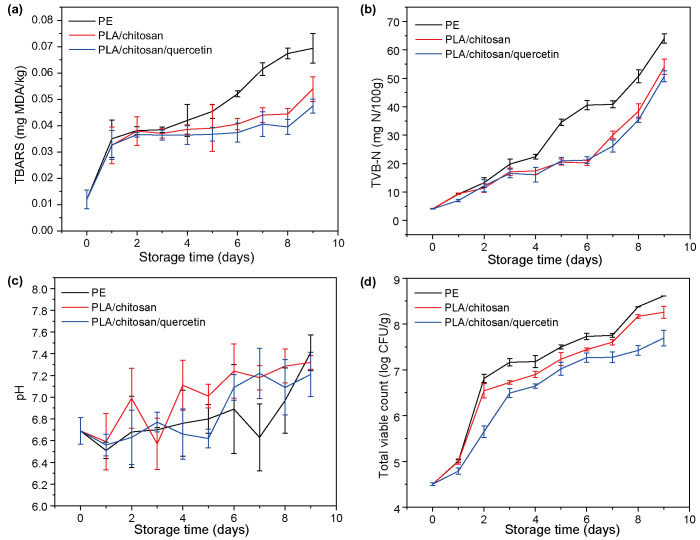
(**a**) TBARS, (**b**) TVB-N, (**c**) pH, and (**d**) TVC values of fresh crucian samples wrapped with the PE, PLA/chitosan, and PLA/chitosan/quercetin films. (PLA: chitosan ratio of 6:3; quercetin concentration was 500 mg/L).

TBARS is important for evaluating the process of fat oxidation in fish. During storage, fish fat was broken down into aldehydes, ketones, and other substances, leading to deterioration of fish quality. Figure 7a illustrates that the TBARS of all three groups gradually increased over time. For the first three days, the TBARS of the fish encapsulated in the PLA/chitosan/quercetin films were only slightly lower than those of the control groups. On the 7th day, the TBRAS values of PE film, the PLA/chitosan film, and the PLA/chitosan/quercetin film were 0.061, 0.044, and 0.040 mg MDA/kg, separately. Until the 10th day, the TBARS values of the fish packaged using PE films reached 0.069 mg MDA/kg, whereas the TBARS of the fish wrapped with the PLA/chitosan and PLA/chitosan/quercetin films remained at 0.054 and 0.047 mg MDA/kg, correspondingly. The TBARS of the fish wrapped with the PLA/chitosan/quercetin film consistently showed lower levels than the control groups across the storage process, probably due to the release of quercetin, which inhibits the partial oxidation of unsaturated fatty acids in crucian [50].

TVB-N content is the quantitative analysis of nitrogenous compounds, including ammonia and organic amines released through microbial metabolism in the spoilage process of animal food [21], which is an essential parameter to evaluate the freshness of fish. Figure 7b shows that the original TVB-N value of all samples remained largely consistent. The TVB-N value of the three groups gradually accumulated over time. On day 6, PE film-packaged fish samples exhibited TVB-N levels of 40.58 mg N/100 g, exceeding the 35 mg N/100 g safety threshold [21]. However, no significant variation was observed in TVB-N value between the fish encapsulated in the PLA/chitosan and PLA/chitosan/quercetin films, with a maximum of only 21.23 mg N/100 g. The observed phenomenon was primarily caused by the antimicrobial properties of PLA and chitosan [21]. The TVB-N of the fish wrapped with the PLA/chitosan and PLA/chitosan/quercetin films reached the limiting values on day 8 of storage, at 38.47 and 35.45 mg N/100 g, respectively. The TVB-N of the fish sample wrapped by the PLA/chitosan film was always slightly higher than that of the sample wrapped by the PLA/chitosan/quercetin film, likely due to the release of quercetin, which inhibited the propagation of bacteria and decreased bacterial capacity for oxidative deamination of compounds [51]. The pH values of the three groups generally decreased and then increased in 10 days (Figure 7c). The decrease in pH from 0 to 1 day may be caused by the breakdown of glycogen or dissolution of CO_2_ in the fish [52]. Subsequently, alkaline components such as ammonia (NH_3_), dimethylamine (DMA), and trimethylamine (TMA) produced during the spoilage of fish led to an increase in pH [21,53].

The microbial colonization was evaluated by measuring the number of bacteria throughout storage. An initial TVC of 4.50 log CFU/g was observed (Figure 7d), suggesting potential low-level microbial contamination during sample preparation. The TVC values increased gradually with time. The International Commission on Microbiological Specifications for Foods (ICMSF) establishes 7 log CFU/g as the maximum allowable TVC for fresh meat [50]. The TVC values of the fish wrapped with PE films reached 7.1 log CFU/g on the 3rd day, exceeding the maximum limit. Therefore, PE film could only maintain the edible quality of fish based on TVC for three days. On the 5th day, the TVC values of samples under the PLA/chitosan and PLA/chitosan/quercetin film-wrapped treatment were 7.23 and 7.02 log CFU/g, correspondingly, which surpassed the upper TVC limit. However, the TVC values of the fish samples encapsulated in the PLA/chitosan/quercetin film were smaller than those of the control group throughout storage, resulting from quercetin’s antimicrobial properties [54]. These findings demonstrate that the freshness retention performance of the PLA/chitosan/quercetin film was the best, followed by the PLA/chitosan composite film, and PE film was the worst. The PLA/chitosan/quercetin film can effectively enhance the freshness of fish and retard fish spoilage by 2 days compared to PE film.

**Figure 8:**
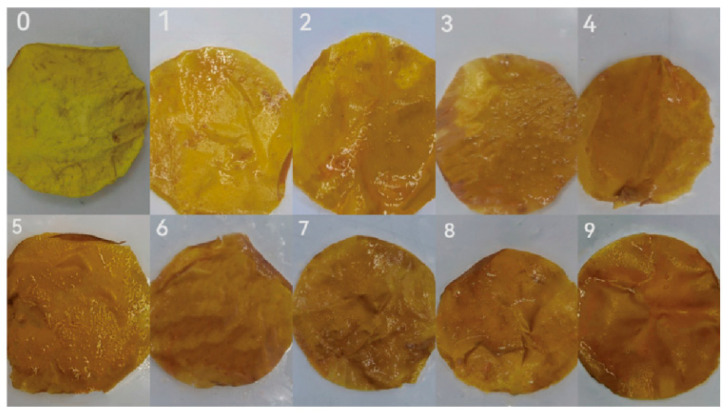
Color change and of the PLA/chitosan/quercetin film in 9 days. (PLA: chitosan ratio of 6:3; quercetin concentration was 500 mg/L).

The storage stability of the PLA/chitosan/quercetin film was first investigated. Color changes in the film stored in the refrigerator over 5 days are shown in Figure S3, indicating that the color of the films remained stable. Next, the application of the PLA/chitosan/quercetin film in fish preservation was studied. Table 1 records the changes in color parameters L*, a*, b*, ∆E, and YI of the film shown in Figure 8. Compared to day 0, both the L* and b* parameters exhibited consistent decreases, whereas the a* value demonstrated a progressive increase, suggesting that the film’s color became darker and redder, with a reduction in yellow components. While the YI value did not show a clear pattern. This may be related to the complex color evolution mechanism of the film during storage. The calculation of YI typically relied on the ratio of b to L, and experimental data showed that while the L* value presented a downward trend, the b* value exhibited some fluctuations during its decline. Their combined changes enhanced the volatility of YI, thus obscuring potential patterns.

The color difference (∆E) basically exhibited an increasing trend, with ∆E close to 30 suggesting deterioration of the fish. For L*, a*, and b*, there were significant differences compared to day 0, further proving that the film effectively reflected the freshness of the fish. These changes are also in line with the pH and TVB-N results in Figure 7. As the freshness of the fish decreases, volatile alkaline substances (DMA, TMA, etc.) are liberated from the fish, boosting the pH, whereas quercetin, as a natural flavonoid, is pH-sensitive. This color change could therefore arise from the capability of volatile ammonium ions to establish alkaline environments on the surface of the film, leading to the deprotonation of the quercetin phenol group, which altered its light absorption characteristics and color [55].

### 3.4. Biodegradability of the Composite Film

**Figure 9:**
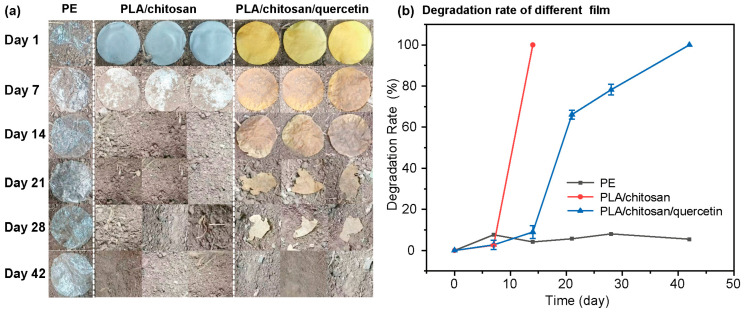
(**a**) Images of the biodegradation of PE film, the PLA/chitosan film, and PLA/chitosan/quercetin films. (**b**) Degradation rate of the films. (PLA: chitosan ratio of 6:3; quercetin concentration was 500 mg/L).

Biodegradability is an important preparation characteristic of food packaging materials, as it directly affects the environmental impact and sustainability of the packaging materials. Microbial activity is the main driving force behind biodegradation, and the pH and moisture content of the soil are important factors influencing microbial activity. Therefore, the soil’s pH was measured at 7.37 ± 0.03, and the moisture content was 9.46 ± 0.38%. The morphological changes in the films over 42 days are shown in Figure 9a. PE film showed no visible changes. In contrast, the PLA/chitosan films were completely degraded by day 14. The PLA/chitosan/quercetin films gradually fragmented from an intact state until they completely disappeared by day 42. Additionally, degradation area percentages were quantified using image software (Figure 9b). The degradation rate of PE film reached only about 5.47% over 42 days. In contrast, the PLA/chitosan film achieved 100% degradation by the 14th day, while the degradation rate of the PLA/chitosan/quercetin film was 8.94% by the 14th day and 78.20% by the 28th day, and it was completely degraded by the 42nd day. This was due to the fact that the ester bonds in the molecular structure of PLA are easily hydrolyzed, which can be metabolized in soil through microbial mediation forming lactic acid, and the final metabolites are water and carbon dioxide [56,57]. Chitosan, as a polysaccharide, can provide an energy source for soil microorganisms, and bacteria in the soil can obtain energy and carbon sources by decomposing chitosan, thereby promoting their growth and reproduction. Quercetin, as a polyphenolic compound with antibacterial properties, may restrict the proliferation of soil microorganisms to a certain extent; so the degradation rate is slower than that of PLA/chitosan composite film.

## 4. Conclusions

In this work, for the purpose of developing biodegradable and active packaging materials, the PLA/chitosan/quercetin film was prepared by taking advantage of the good compatibility of chitosan and PLA, along with the superior antimicrobial and antioxidant activities of quercetin. FTIR confirmed the physical bonding of quercetin with chitosan and PLA. The introduction of quercetin maintained the water vapor permeability and polymer matrix continuity of the PLA/chitosan film while reducing the oxygen permeability of the composite film from 2.772 × 10^−3^ cm^3^/(m^2^·d·Pa) to 1.416 × 10^−3^ cm^3^/(m^2^·d·Pa). It also improved the hydrophobicity and simultaneously enhanced the antimicrobial properties, providing more possibilities to meet diverse packaging requirements. In addition, the PLA/chitosan/quercetin film can be completely degraded within 42 days, minimizing environmental residue risks compared to traditional petroleum-based packaging materials. The PLA/chitosan/quercetin film has a certain inhibitory effect on microbial growth and protein lipid oxidation. Shelf life tests demonstrated that the PLA/chitosan/quercetin film could retard fish spoilage by 2 days compared to PE film, and preservation efficacy was better than that of the PLA/chitosan film, attributing to the strong antioxidant and antimicrobial ability of quercetin. Moreover, according to quercetin’s pH sensitivity, the PLA/chitosan/quercetin film can also respond to the change in fish freshness in real time, which is promising for the application in active smart packaging, providing a more efficient and sustainable guarantee for food preservation and visualization monitoring.

## Figures and Tables

**Table 1:** Color parameters analysis of PLA/chitosan/quercetin film in 9 days.

**Day**	L*	a*	b*	△E	YI
0	53.40 ± 1.14 ^b^	2.20 ± 0.45 ^g^	53.80 ± 0.84 ^a^		143.97 ± 3.05 ^ab^
1	61.00 ± 1.00 ^a^	5.40 ± 0.55 ^f^	55.60 ± 0.89 ^a^	11.01 ± 0.89 ^f^	130.23 ± 2.52 ^c^
2	50.80 ± 0.84 ^c^	10.40 ± 0.89 ^e^	51.00 ± 1.00 ^b^	13.25 ± 1.33 ^e^	143.45 ± 3.70 ^ab^
3	42.40 ± 1.14 ^d^	11.60 ± 0.89 ^d^	39.40 ± 1.14 ^d^	22.81 ± 1.43 ^d^	132.76 ± 2.26 ^c^
4	38.40 ± 0.89 ^e^	12.00 ± 0.71 ^d^	39.80 ± 1.48 ^cd^	25.03 ± 1.70 ^c^	148.20 ± 8.09 ^a^
5	44.00 ± 1.58 ^d^	13.00 ± 1.00 ^c^	41.60 ± 1.14 ^c^	21.74 ± 1.34 ^d^	135.26 ± 7.14 ^bc^
6	36.00 ± 1.58 ^f^	13.80 ± 1.10 ^bc^	34.20 ± 2.77 ^e^	30.84 ± 1.30 ^a^	136.05 ± 13.76 ^bc^
7	38.20 ± 2.17 ^e^	14.20 ± 0.84 ^b^	36.20 ± 1.92 ^e^	28.52 ± 2.66 ^b^	135.49 ± 5.74 ^bc^
8	38.20 ± 2.17 ^e^	15.40 ± 0.55 ^a^	35.60 ± 1.52 ^e^	29.59 ± 2.48 ^ab^	133.36 ± 6.78 ^c^
9	34.80 ± 2.59 ^f^	16.20 ± 0.45 ^a^	36.00 ± 1.22 ^e^	31.69 ± 1.35 ^a^	148.20 ± 7.61 ^a^

Note: Different superscript letters indicate significant differences (*p* < 0.05).

## Data Availability

The original contributions presented in this study are included in the article/Supplementary Material. Further inquiries can be directed to the corresponding authors.

## Supplementary Materials

The following supporting information can be downloaded at: https://www.mdpi.com/article/10.3390/foods14162771/s1, Figure S1. Photos of fish preserved with PE, PLA/chitosan, and PLA/chitosan/quercetin films; Figure S2. Bacterial counts of *E. coli* and *S. aureus* in the liquid culture medium without film (control) and liquid culture medium treated with the PLA/chitosan film or the PLA/chitosan/quercetin film for 24 h (PLA: chitosan ratio of 6:3; quercetin concentration was 500 mg/L); Figure S3. Color changes in the PLA/chitosan/quercetin film stored in the refrigerator (4 °C) over 5 days, along with ΔE and YI analysis.
