# Supplementary material for: Biodegradable Quercetin-Incorporated Poly(Lactic Acid)/Chitosan Functional Films: A Study of the Properties and Application in Enhancing Fish Preservation
Source: Foods. 2025 Aug 9;14(16):2771. doi: 10.3390/foods14162771 (PMC12385204; doi:10.3390/foods14162771)
Supplement: Supplementary file 1 [file foods-14-02771-s001.zip › foods-3749509-supplementary.pdf]

Supplementary Data

2.3.6 Antibacterial activity

Colonies of *E. coli* and *S. aureus* were separately picked from slant culture media and placed in beef extract and peptone liquid media to activate for 24 h, then diluted 10^3 times with saline. The PLA/chitosan film and PLA/chitosan/quercetin film were cut into circular samples with 6 mm diameter (three replicates per group), sterilized on both sides under UV light for 30 min, and immersed in bacterial suspensions. A suspension without film served as a blank control. Samples were incubated with shaking at 37 °C and 200 rpm for 24 h. The reacted bacterial solution was diluted 10^4 times, and 100 μL of the diluted solution was spread on a solid culture medium. The samples were incubated at 37 °C in a constant temperature incubator for 15 h, followed by photographing and counting the colonies on each culture medium.

2.4. Antioxidant activity of the PLA/chitosan/quercetin film

A 7 mM ABTS solution was prepared by adding 10 mg ABTS to 2.6 mL of 2.45 M potassium persulfate. The reaction was carried out in a dark environment at room temperature for 12-16 h. 1 mL ABTS solution was taken and diluted 100 times with pure water to achieve an absorbance of 0.7±0.02 at 734 nm. The absorbance of the diluted solution was recorded as A_0 (blank). The sample film was placed in 3 mL ABTS diluted solution, and reacted at room temperature for 1 h. Then the film was removed, and the absorbance of the reaction solution was measured and recorded as A_1.

2.5.4. TVB-N

Firstly, 0.01 mol/L hydrochloric acid titration solution and 20 g/L boric acid solution were prepared. 1 g/L Methyl red and 1 g/L bromocresol green were mixed in a 1:5 ratio, and boric acid was mixed with the combined indicator in a 100:1 ratio. The prepared solutions were placed into the automatic Kjeldahl nitrogen determination apparatus (Hanon Instruments K1160, China). Then 25 mL of ultrapure water was added to 5 g fish and soaked for 30 min. 20 mL of the immersion liquid and 1 g of MgO powder was added to the reaction tube, after which the testing began and the TVB-N values were recorded.

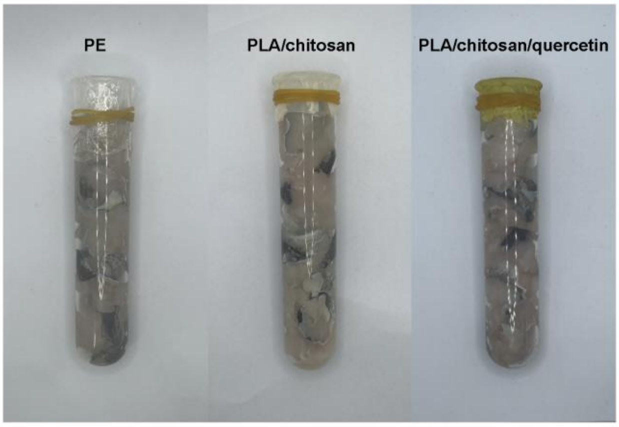

Figure S1. Photos of fish preserved with PE, PLA/chitosan, and PLA/chitosan/quercetin films.

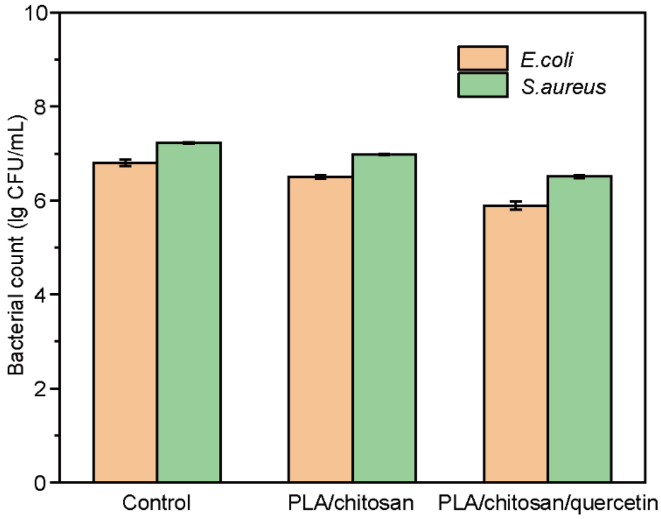

Figure S2. Bacterial counts of *E. coli* and *S. aureus* in the liquid culture medium without film (control) and liquid culture medium treated with the PLA/chitosan film or the PLA/chitosan/quercetin film for 24 h (PLA: chitosan ratio of 6:3; quercetin concentration was 500 mg/L).

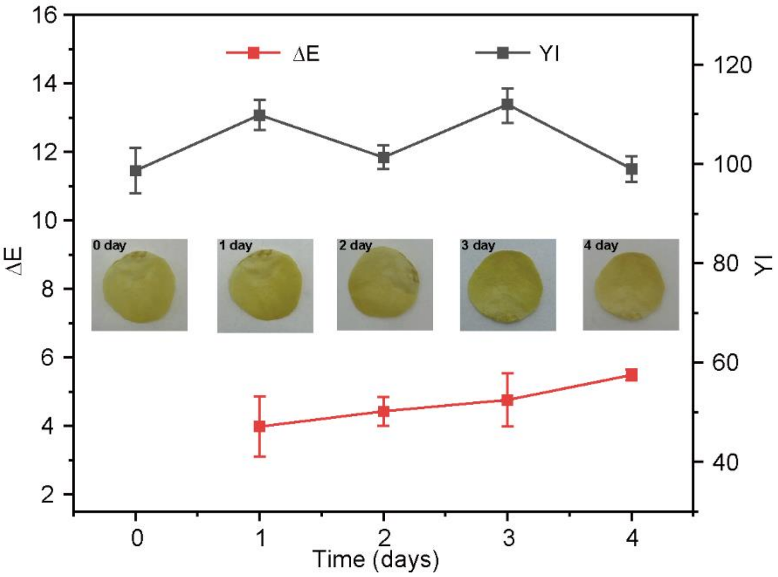

Figure S3. Color changes of the PLA/chitosan/quercetin film stored in the refrigerator (4 °C) over 5 days, along with ΔE and YI analysis.
